# Editorial Notes

**Published:** 1883-11

**Authors:** 


### EDITORIAL NOTES.

The Colleges and the Licensing Power.—The *New York Medical Journal* says that the Medical Society of the County of Westchester has lately taken the decided step of preparing a memorial addressed to the legislature, praying that body to take away from the medical colleges the power to grant licenses to practice, and practically to give that power to the Board of Regents of the University of the State of New York. Such action, of course, would reduce the colleges to mere teaching bodies. This would likewise be the result in case the bill drawn up by the Erie county society should become a law. From the fact that the Westchester society has addressed a communication on the subject to our own society, it may be inferred that concerted action on the part of the various county societies is to be attempted.

Dr. Bulkley will give a seventh course of lectures on "Diseases of the Skin," in the Pathological Amphitheatre of the New York Hospital, 7 West 15th street, Wednesday afternoons at 2.30 o'clock, commencing Wednesday, October 17, 1883 The lectures will be didactic and clinical in character, covering the entire subject of diseases of the skin (including syphilis) and will be freely illustrated by colored plates, photographs, life-sized models, the blackboard, and abundant clinical material. The pathology, differential diagnosis and treatment of diseases of the skin will be especially considered. The course will consist of twenty lectures and will be free to practitioners of medicine and medical students.

The U. S. Pharmacopœia.—We are requested by the publishers, Messrs Wm. Wood & Co., 56 and 58 Lafayette Place, New York, to announce that any person having purchased a copy of the U. S. Pharmacopœia of 1880, and desiring a list of the corrections since made therein, can procure the same by sending a two-cent stamp to them.

A Gold Medal to W. H. Schieffelin & Co, of New York.—We call attention to the following extract from the New York *Medical Record*, as an instance of a well-earned honor: "At the Holland International Exhibition, at Amsterdam, a Gold Medal has just been awarded to Messrs. W. H. Schieffelin & Co. for their Soluble Pills and Granules. This is a special distinction for their reliable preparations, as the award was made after a careful analysis of specimens of Messrs. Schieffelin's manufactures by prominent chemists."

We call attention to the advertisement of Henry Herrmann, 322 E. Genesee street, who is a practical instrument maker, and also repairs and puts in order surgeons' and physicians' cases. We commend him to the profession for their patronage and convenience.

THE aggressiveness of American enterprise received a very striking illustration at the late International Pharmaceutical Exhibition held at Vienna, in the display of products from the laboratory of our energetic countrymen, Messrs. Parke, Davis & Co., of Detroit, Michigan. We notice in the reports as published in the domestic journals, and from the special correspondence of foreign journals, that this display, while exciting much interest from its scientific features, attracted more than ordinary notice from its artistic beauty and finish, among the non-professional visitors. This lay interest was, doubtless, largely due to the special attention given the display by the Emperor and the Archduke Karl Ludwig. These royal visitors manifested unusual interest in this exhibition of American taste, and took occasion to especially compliment the firm; through its representative, on its enterprise and skill. We congratulate Messrs. Parke, Davis & Co., on this distinguished recognition of the artistic excellence of their laboratory products. Their intrinsic worth needs no commendation from us; this has long been conceded by the profession. The gold medal awarded them by the Vienna Exhibition is but an endorsement of the esteem in which this scientific commercial house is held in this country, where it is best known.

